# VIJB: a companion of the JBROWSE genome browser for the visually impaired people

**DOI:** 10.1093/bioinformatics/btag396

**Published:** 2026-06-16

**Authors:** Salomé Nashed, Patricia Uguen, Laurent M Sachs, Nicolas Buisine

**Affiliations:** Unité Mixte de Recherche 7221, Département Adaptation du Vivant, Centre National de la Recherche Scientifique, Muséum National d‘Histoire Naturelle, Alliance Sorbonne Universités, Paris, France; Unité Mixte de Recherche 3348, Centre National de la Recherche Scientifique, Institut Curie, Université Paris-Saclay, Orsay, France; Unité Mixte de Recherche 7221, Département Adaptation du Vivant, Centre National de la Recherche Scientifique, Muséum National d‘Histoire Naturelle, Alliance Sorbonne Universités, Paris, France; Unité Mixte de Recherche 7221, Département Adaptation du Vivant, Centre National de la Recherche Scientifique, Muséum National d‘Histoire Naturelle, Alliance Sorbonne Universités, Paris, France; Unité d’Appui et de Recherche 2047, Centre National de la Recherche Scientifique, Muséum National d‘Histoire Naturelle, Alliance Sorbonne Universités, Paris, France

## Abstract

**Motivation:**

The availability of touch-sensitive and haptic devices has been a keystone development for the inclusion of visually impaired people (VIPs) in modern, highly digitized work environments. Braille displays have proven efficient and versatile enough to parse large and complex text files, making bioinformatics and text-heavy programming accessible to VIPs. However, the complex graphical objects -combining numerous datasets- typically generated during data integration remain challenging, even with the aid of descriptive AI. This is particularly true in functional genomics. Here, we present VIJB, a simple application that displays the multilayered output of the JBROWSE genome browser on a Braille reader, enabling VIPs to fully participate in data integration in functional genomics.

**Availability and Implementation:**

VIJB is programmed in Python and relies on the scientific library NumPy, the braillegraph and pyBigWig libraries, and the TABIX software. The architecture is summarized in Supplementary Material 1, available as supplementary data at *Bioinformatics* online. VIJB is available for download at the GitHub repository https://GitHub.com/NiBuMNHN/VIJB and is licenced under the GPL 3.0.

## 1 Introduction

Genome browsers are software tools dedicated to the visual integration of structural and functional data along DNA. This includes both qualitative data (e.g. gene and transcript annotations, transposable elements, CpG islands) and quantitative data (e.g. recombination rates, RNA expression, protein enrichment). With the constant advances in DNA and RNA sequencing technologies, genome browsers have quickly become essential components of modern biology. A significant part of their success stems from their ease of use: users typically need only select the tracks to be displayed from a list and specify the genomic location of interest. The image generated on-the-fly offers a quick and intuitive way to integrate large and complex datasets.

Genome browsers have been developed as web services, desktop applications (Kent *et al*. 2002, Stein 2013, Thorvaldsdóttir *et al*. 2013), and sometimes as both (Diesh *et al*. 2023). Desktop solutions are relatively easy to set up and use by non-bioinformaticians, and they offer the possibility to work offline. However, such solutions may not be fully effective given the large size of datasets often generated during a project and the limited hard drive space of desktop computers and laptops. Additionally, the dispersion of datasets across numerous local machines can quickly become a source of confusion, as they may not all be synchronized with each other. When available as web services, genome browsers such as GBROWSE and JBROWSE act as centralized information systems, making efficient use of hard drive space and limiting data replication, but at the cost of depending on a system administrator.

Typically, after selecting the tracks of interest and the genomic location, genome browsers represent the genomic features of a locus as a stack of tracks, each corresponding to an annotation layer or quantitative data, displayed through histograms or heatmaps. For very large genomic regions (e.g. 1 Mb), the graphical features are compressed to fit the image width (e.g. 1024 pixels).

Historically, the GMOD genome browser (GBROWSE) has been a popular companion for genomic projects and has been instrumental in major initiatives. This application relied heavily on Perl-CGI, AJAX, BioPerl, and stored data in a relational SQL database, typically hosted on a LAMP server (Linux, Apache, MySQL, PHP). Displaying quantitative tracks in BigWig format required the installation of additional software available from UCSC *et al.* 2002. Overall, the installation procedure was complex, and the ever-increasing size of databases impacted performance.

JBROWSE is a modern genome browser based on the Node.js implementation of JavaScript. It is fast, reliable, and relatively easy to install. Unlike GBROWSE, data in JBROWSE are accessible through a set of compressed and indexed flat text files. Metadata (e.g. the location of data files, their format) are documented in a JSON-formatted database. By design, JSON is a text-based open standard designed for human-readable data interchange. Therefore, data source and track configurations are accessible both programmatically and manually.

Visually impaired persons (VIPs) working in biology have very limited access to experimentation. However, in principle, they can more easily develop careers in bioinformatics and other digital processing fields. While most people are comfortable using feature-rich graphical user interfaces (GUIs) of modern operating systems, these are harder to manipulate for VIPs (Boulton 1993, O’Modhrain *et al.* 2015, Pundlik *et al.* 2023). Instead, data flow in a terminal or text editor is more linear and practical for VIPs. Haptic devices, such as Braille readers, efficiently convert simple text and thus make the practice of bioinformatics accessible. To date, accessible visualization tools for genome features do not exist.

More generally, active research on haptic devices and multisensory feedback aims to deepen engagement with digital environments, enabling data visualization, enhanced interpretation of complex data, improved learning and training, and safe experimentation (Reiner 2008, Paneels and Roberts 2010; See, Choco and Chandramohan 2022, Tan *et al.* 2025). Despite an increasing trend, initiatives around accessible science training for VIPs remain very limited (Ariza and Hernández Hernández 2025, Petitdemange and Nashed 2025), and only a few VIPs pursue scientific careers.

We present VIJB, a helper application for the JBROWSE genome browser aimed at VIPs. The name VIJB (for *Visually Impaired JBROWSE*) also evokes the GUI-less VI text editor found in most Unix/Linux systems. This program is designed to access a JBROWSE-compliant JSON-formatted database, collect the corresponding data, and translate them for display on a Braille reader, thereby making the visualization of genomic features accessible to VIPs. Indirectly, reducing the technical obstacles between visually capable researchers and VIPs smooths workflows, promotes inclusion, and fosters team spirit. VIJB can also be used by sighted individuals seeking terminal-only alternatives to genome browsers.

## 2 Implementation

VIJB is designed to run alongside the web version of JBROWSE, directly interrogating its database and displaying data on a Braille reader. The output is a black-and-white (pins down or up) image to navigate through and does not correspond to a conventional set of Braille characters.

The database is stored as a JSON file (*config.json*), located at the root of the JBROWSE folder hosting the application. This file indicates every track’s metadata, individual file locations, and their file formats. In principle, as long as it follows the same schema, VIJB can interrogate any JSON database, even if JBROWSE is not installed. VIJB runs on macOS, Windows (with the Linux subsystem WSL), and Linux, although the limited vocal synthesis capabilities for describing screen content on Linux make it less user-friendly for daily use.

The overall architecture of VIJB is described in Supplementary Material 1, available as supplementary data at *Bioinformatics* online, with a detailed schematic and accessible legend. VIJB is written in Python and requires the json library and the scientific library NumPy. Data access is similar to that of JBROWSE: annotation files in GFF3 or BED format must be bgzip-compressed and indexed with TABIX (Li 2011). The Braille reader relies on the braillegraph library (https://github.com/chrisbouchard/braillegraph), and extracting data from compressed BigWig-formatted files uses the pyBigWig library (Zhang *et al*. 2020).

When used alongside JBROWSE, VIJB should be installed on the same server, and the database (*config.json*) and data files must have read permissions. VIJB can be installed either in a user’s home folder or in a system-wide folder (e.g. /*usr/bin/*) to make it available to all users. Users then need to access the server through SSH.

Braille readers are available in different widths, the most typical being 80 dots (40 columns with 2 dots per column) long and 8 dots in height. By default, VIJB output is configured for this setup, but this can be adjusted using dedicated options. Braille readers also have buttons to scroll horizontally when lines exceed 40 Braille columns in length. This can be useful to increase the display resolution. In such cases, it may also be beneficial to adjust the column width of the output.

Due to the limited “resolution” of Braille readers (typically 8 × 80 columns) compared to a full-featured 800 × 1024-pixel image, the output of each track is averaged within windows and mapped to a reduced representation using the NumPy interpolation function *interp*. A similar situation, albeit less dramatic, exists with JBROWSE when the displayed genome span is larger than the image width (e.g. a 1 Mb region on a 1024-pixel-wide image).

## 3 Usage

VIJB features a simple command interpreter that works in conjunction with options. The command vocabulary is limited but can be easily expanded. Commands (*assemblies*, *tracks*, and *display*) are used to specify the type of task: listing available genome assemblies, providing the list of all tracks, and displaying genomic data, respectively. Options are used to set environment parameters and specify the genomic location of interest. The *-h* option displays a brief help message summarizing the available commands and options.

A call to the *display* command results in a single track being displayed on the Braille reader. It is easy to mimic the JBROWSE display—featuring multiple tracks stacked on top of each other—using a simple shell script that automates multiple calls to VIJB (Supplementary Material 2, available as supplementary data at *Bioinformatics* online). The “up” and “down” buttons on the reader then allow users to navigate through the tracks.

The supported format for quantitative tracks is BigWig. Density profiles are displayed as histograms, followed by additional statistics (minimum and maximum values) for the region being displayed. There is also a “raw mode” (“*-r”* option), in which numerical values are displayed instead of histograms.

For annotation tracks (e.g. gene transcripts, repetitive sequences, or other box-like features), the supported formats are GFF3 and BED. Both offer simple ways to represent complex genomic features composed of sub-features. GFF3 provides additional flexibility, as sub-features can be encoded on different strands, which is often necessary to represent PCR primer pairs or ChIA-PET long-range interaction clusters (Fullwood *et al*. 2009, Buisine *et al*. 2015). When the strandness option (“*-s”*) is invoked, each track is split into top and bottom strands.

Sub-feature aggregation into a single model (e.g. a gene composed of exons connected to each other) is naturally encoded in BED format. VIJB can display them as multiple sub-features connected together or as a single box encompassing the entire genomic range of the feature. In contrast, the GFF3 encoding of sub-features is more complex, involving independent entries (one for each sub-feature) connected to a “master” entry through a “Parent” tag. This complexity allows the encoding of multi-layered information, such as genes or split-transposable elements. In VIJB, parent-child relationships are controlled by specifying the GFF3 fields *“source”* and *“type”* for the sub-features (child, “*-u”* and “*-v”* options) and the aggregator (parent, “*-p”* and “*-q”* options), even if the relationship is not direct and spans multiple levels. This allows full flexibility in displaying the desired level of detail. For genes, the “*—gene”* option automatically sets “*-v”* to *exon* and “*-q”* to *gene*. For convenience, a companion Python script (*vijb_GFF_source_type.py*) describes the *“source”* and *“type”* combinations found in a GFF3 file.

Despite its flexibility, the GFF3 format has evolved into numerous dialects. Here, we follow the standard GFF3 specifications from (https://github.com/The-Sequence-Ontology/Specifications/blob/master/gff3.md).

There are two visualization modes, equivalent to JBROWSE’s “compact” and “extended” views. In the “compact” mode (default), all features are displayed in a single window, whereas in the more detailed “extended” view (“*-x”* option), each aggregated feature is displayed in its own window.

To help users familiarize themselves with the Braille output, VIJB includes a dataset with peaks scaled to display low (0 to 5), medium (0 to 50), and high (0 to 300) dynamic ranges.

## 4 User feedback

Collecting feedback from VIPs is challenging due to their under representation in science, particularly in functional genomics. Nevertheless, VIJB has been tested by a postdoctoral researcher in functional genomics (S. Nashed) and a small group of VIPs, including a biology student, a physics researcher, a law graduate student, and a few high school students. All participants used macOS and Windows WSL with Braille displays from different brands. The installation procedure and command-line interface were found to be flawless and straightforward to use. The ability to flip graphs vertically or horizontally (“-z” option) proved to be a useful feature for building mental images, as mental representations differ between individuals who were affected at birth and those who lost their sight later in life.

Interpreting graphs is a new experience that requires training. To address this, datasets with varying peak heights are provided online, along with accessible examples in raw text format (README.md). The fact that VIJB and JBROWSE interrogate the same databases will also facilitate training and cooperation between VIPs and sighted people in teams.

The pin resolution of the graphs displayed on Braille readers is already close to the maximum level of detail distinguishable by fingertips. Therefore, increasing the device resolution would not be beneficial. Instead, the ability to zoom in and out by adjusting the genome span of the region of interest is a practical solution for achieving the desired level of detail.

## 5 Usage example

A simple shell script querying a gene annotation track and an RNA-Seq quantitative tracks could be:

#!/usr/bin/env bash

LOC=“chr6:356183.356912”

python3 ./vijb.py -z -n RefGenes—gene -g“$LOC” display

python3 ./vijb.py -z -n control -g “$LOC” display

The output, which displays exons connected by a thin line, and RNA-Seq peaks is:


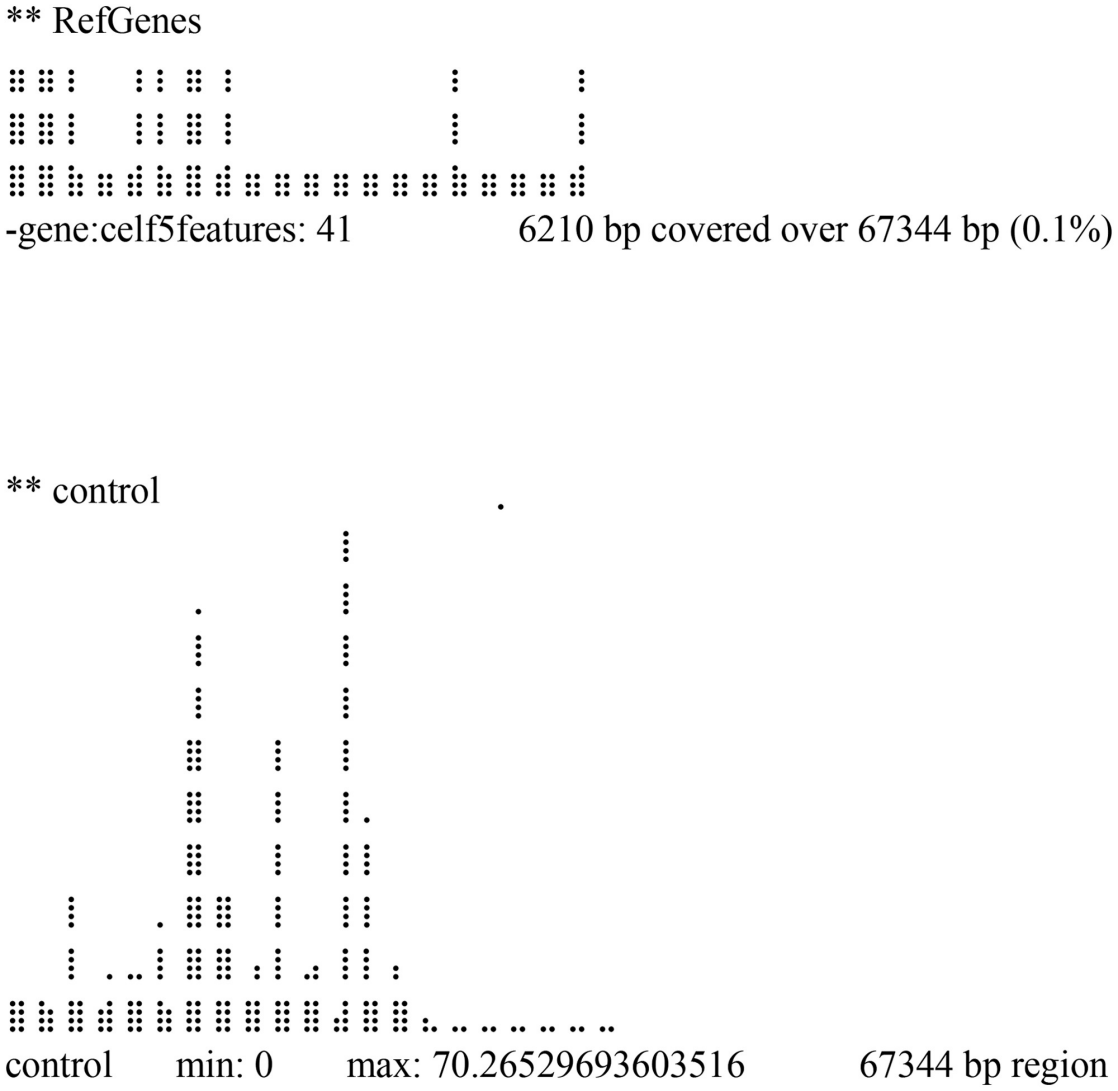


## Supplementary Material

btag396_Supplementary_Data

## Data Availability

There are no new data associated with this article.
