# Supplementary material for: VIJB: a companion of the JBROWSE genome browser for the visually impaired people
Source: Bioinformatics. 2026 Jun 16;42(7):btag396. doi: 10.1093/bioinformatics/btag396 (PMC13372686; doi:10.1093/bioinformatics/btag396)
Supplement: btag396_Supplementary_Data [file btag396_supplementary_data.pdf]

Supplementary material 1: VIJB architecture.

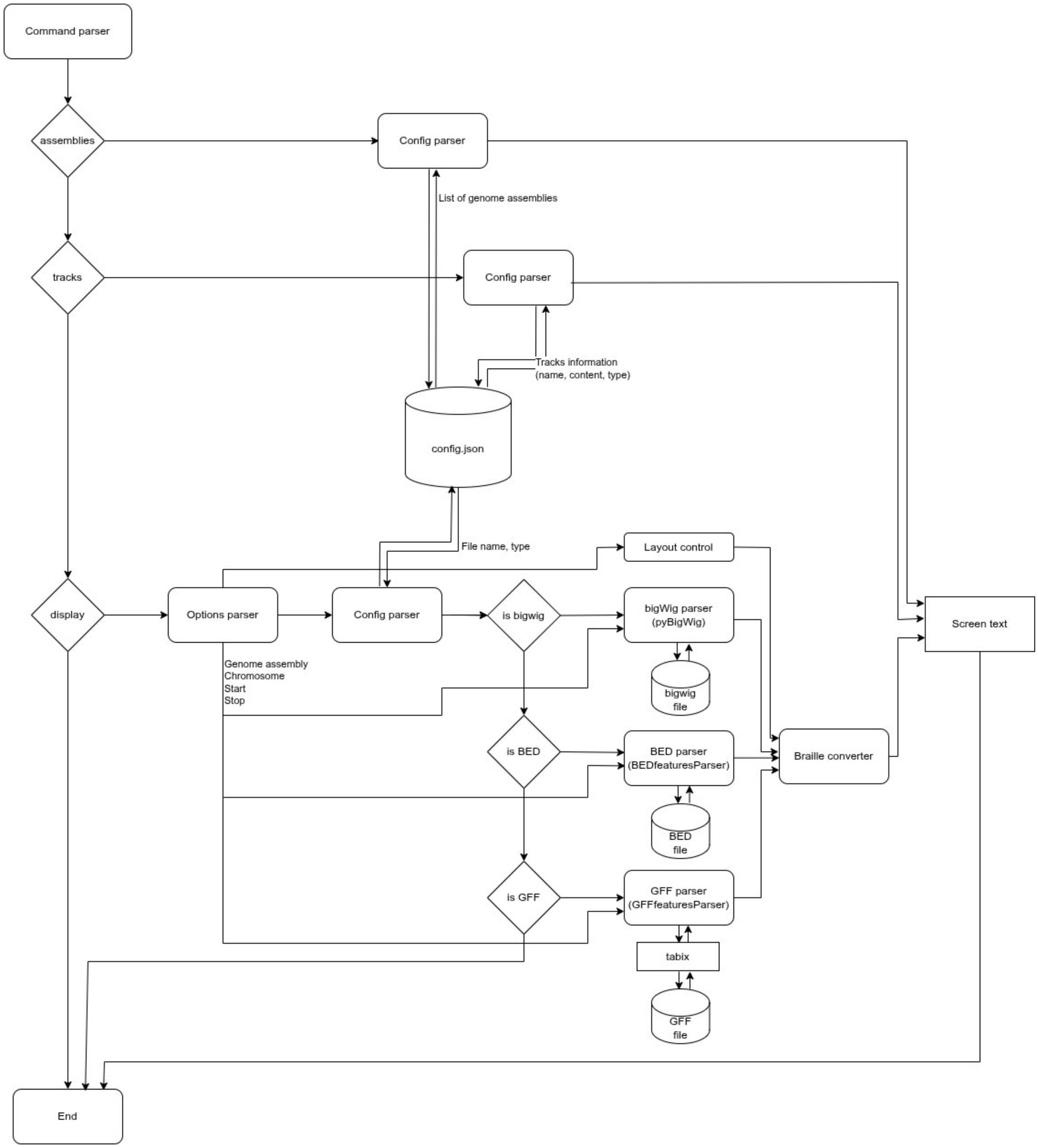

Supplementary material 1: VIJB architecture.

The minimum information required to display a track includes the assembly name, genomic coordinates, and track name. To this end, VIJB implements a simple command interpreter that interrogates the JBROWSE database (or any equivalent JSON database). The available commands are:

- "assemblies" – lists available assemblies;
- "tracks" – provides a detailed list of available tracks (including assembly, data type, name, etc.);
- "display" – builds a reduced representation of a track for display on a braille reader.

The type of track requested is automatically extracted from the JSON database, and data are retrieved from individual files using specific connectors (pyBigWig for BIGWIG files, BEDfeaturesParser for BED files, and GFFfeaturesParser for GFF3 files). Once built, the graph is sent to the Braille converter. The output is formatted according to command-line options before being sent to standard output (e.g., screen or Braille reader). Displaying a stack of tracks is emulated through multiple calls to VIJB.

Supplementary material 2: Use cases.

Important:
Braille characters should be interpreted not as individual symbols but as groups of dots forming graphical representations of genomic features and quantitative signals. Feature representation (e.g., genes, exons) is configurable: genes can be displayed either as a continuous box without internal structure (showing only gene boundaries) or with their exon-intron structure explicitly shown. In the latter case, exons are displayed as small boxes connected together with a faint line at the bottom of the track (controlled by the -u and -v options).

The command “`python3 vijb.py tracks`” returns all tracks information in a tabulated format. In this example, the track named Genes if a GFF file located in data/annot.sorted.gff.gz (relative to the location of config.json).

type	FeatureTrack			
trackId	annotation.gff			
name	Genes			
adapter				
	type	Gff3TabixAdapter		
	gffGzLocation			
		uri	data/annot.sorted.gff.gz	
		locationType	UriLocation	
	index			
		location		
			uri	data/annot.sorted.gff.gz.tbi
			locationType	UriLocation
		indexType	TBI	
category	['Genome_annotation']			
assemblyNames	['genome']			

type	QuantitativeTrack		
trackId	control		
name	control		
adapter			
	type	BigWigAdapter	
	bigWigLocation		
		uri	./data/Control.bw
		locationType	UriLocation
category	['Functiona	genomics']	
assemblyNames	['genome']		
description	control		

type	QuantitativeTrack		
trackId	treatment		
name	treatment		
adapter			
	type	BigWigAdapter	
	bigWigLocation		
		uri	./data/Treatment.bw
		locationType	UriLocation
category	['Functiona	genomics']	
assemblyNames	['genome']		
description	treatment		

* Test datasets
Sample test files are composed of:
- config.json, a minimally configured JBrowse-compliant JSON configuration file,
- data/Control.bw and data/Treatment.bw, BigWig files encoding quantitative measures of gene expression,
- data/control.expanded.bw and data/treatment.expanded.bw, scaled up versions of previous files,
- data/control.flat.bw and data/treatment.flat.bw, scaled down versions of previous files,
- data/annot.sorted.gff.gz and data/annot.sorted.gff.gz.tbi: sample compressed GFF gene annotation, and tabix index file.
(Methylome datasets SRR15206307 and SRR15206306 from Buisine et al., 2021. doi: 10.3390/cells10092375)

* Installation testing

For installation testing, simply run the following command:

```
bash sample_test.sh
```

The expected output is the following:

** Genes

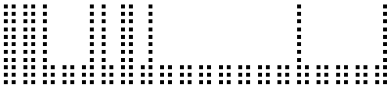

-gene:celf5 features: 41   6210 bp covered over 67344 bp (0.1%)

** Genes

Plus strand

Minus strand

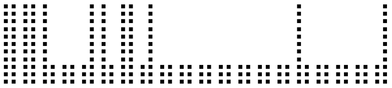

-gene:celf5 features: 41   6210 bp covered over 67344 bp (0.1%)

** Genes

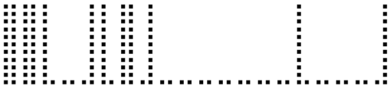

exon-:exon-NM_001097170.1-13,exon-XR_001924017.2-7,exon-XR_0042218... features: 41   6210 bp covered over 67344 bp (0.1%)

** control

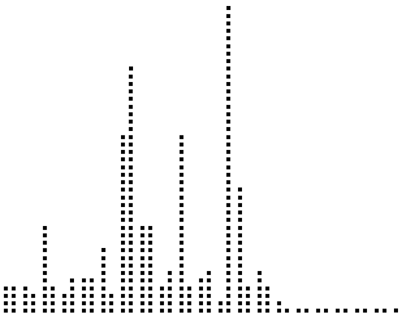

control   min: 0   max: 70.26529693603516 67344 bp region

** treatment

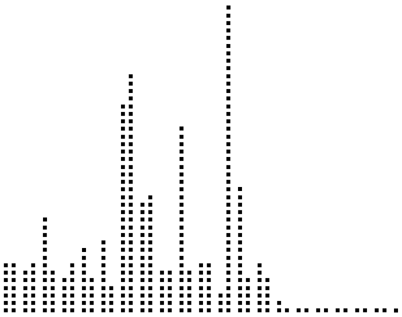

```
treatment   min: 0      max: 52.05149841308594  67344 bp region
```

* Training

To help visually impaired users build mental images of the graphs, test datasets are also available in "flattened" (minimal peak height) and "expanded" (maximal peak height) formats. Although peak shapes may appear similar, users should refer to the annotation/summary line at the bottom of each track, which indicates the maximum peak height and can reveal substantial differences between actual density profiles.

Notice the differences in scale compared to the bottom line of each track, despite peaks that may look similar. Two types of information can be read from such a graph:

- the **profile**, i.e., the shape of the peaks;
- their **amplitude**, or peak height.

The output of the script `"training_test.sh"` is shown below:

** Genes

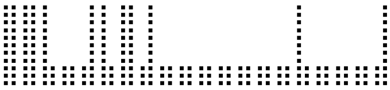

```
-gene:celf5 features: 41      6210 bp covered over 67344 bp (0.1%)
```

** control

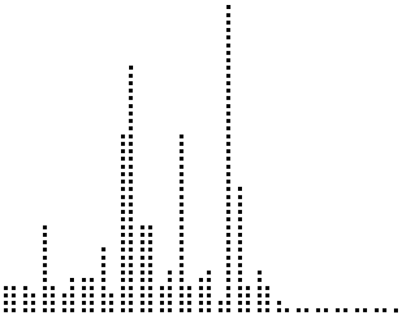

```
control     min: 0      max: 70.26529693603516  67344 bp region
```

** treatment

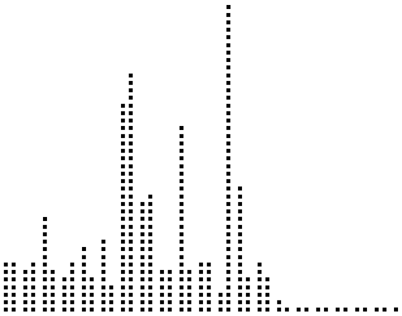

treatment   min: 0   max: 52.05149841308594 67344 bp region

** control_expanded

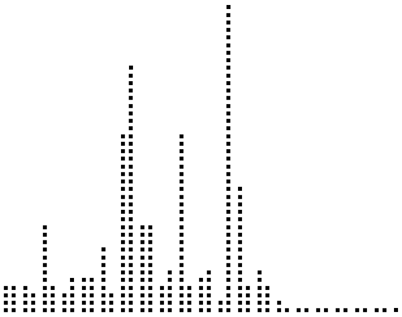

control_expanded min: 0   max: 351.3269958496094 67344 bp region

** treatment_expanded

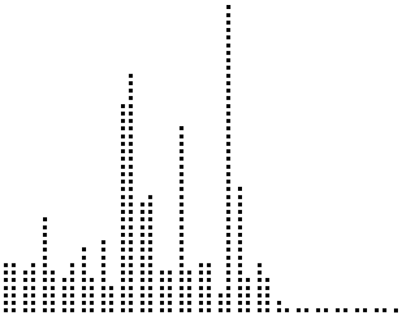

treatment_expanded   min: 0   max: 260.2569885253906 67344 bp region

** control_flat

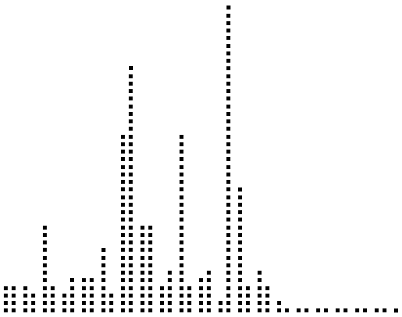

control_flat   min: 0   max: 7.026529788970947 67344 bp region

** treatment_flat

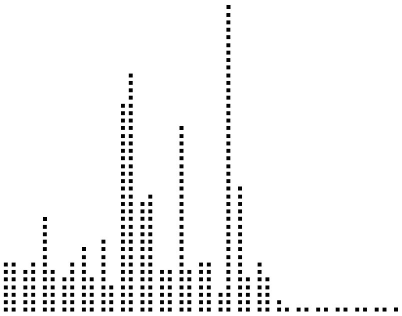

treatment_flat   min: 0   max: 5.205150127410889 67344 bp region
